# Immunohistochemical and Ultrastructural Study of the Degenerative Processes of the Hip Joint Capsule and Acetabular Labrum

**DOI:** 10.3390/diagnostics15151932

**Published:** 2025-07-31

**Authors:** Riana Maria Huzum, Bogdan Huzum, Marius Valeriu Hînganu, Ludmila Lozneanu, Fabian Cezar Lupu, Delia Hînganu

**Affiliations:** 1 Department of Radiology, Faculty of Medicine, “Grigore T. Popa” University of Medicine and Pharmacy, 400347 Iasi, Romania; riana-maria.huzum@umfiasi.ro; 2 Department of Orthopedics and Traumatology, Faculty of Medicine, “Grigore T. Popa” University of Medicine and Pharmacy, 400347 Iasi, Romania; bogdan.huzum@umfiasi.ro; 3 Department of Morpho-Functional Sciences I, Faculty of Medicine, “Grigore T. Popa” University of Medicine and Pharmacy, 700115 Iasi, Romania; ludmila.lozneanu@umfiasi.ro (L.L.); hinganu.delia@umfiasi.ro (D.H.); 4 Department of Mechanical, Mechatronics and Robotics Engineering, Mechanical Engineering Faculty, “Gheorghe Asachi” Technical University of Iasi, 700050 Iasi, Romania; fabian-cezar.lupu@academic.tuiasi.ro

**Keywords:** hip capsule, acetabular labrum, osteoarthritis, immunohistochemistry, SEM, SOX9, CD68, ERG, lubricin, joint degeneration

## Abstract

**Background/Objectives**: Degenerative processes of the hip joint increasingly affect not only the articular cartilage but also periarticular structures such as the joint capsule and acetabular labrum. This study aimed to investigate the structural and molecular changes occurring in these tissues during advanced hip osteoarthritis. **Methods**: A combined analysis using immunohistochemistry (IHC), scanning electron microscopy (SEM), and micro-computed tomography (microCT) was conducted on tissue samples from patients undergoing total hip arthroplasty and from controls with morphologically normal joints. Markers associated with proliferation (Ki67), inflammation (CD68), angiogenesis (CD31, ERG), chondrogenesis (SOX9), and lubrication (Lubricin) were evaluated. **Results**: The pathological group showed increased expression of Ki67, CD68, CD31, ERG, and SOX9, with a notable decrease in Lubricin. SEM analysis revealed ultrastructural disorganization, collagen fragmentation, and neovascular remodeling in degenerative samples. A significant correlation between structural damage and molecular expression was identified. **Conclusions**: These results suggest that joint capsule and acetabular labrum degeneration are interconnected and reflect a broader pathophysiological continuum, supporting the use of integrated IHC and SEM profiling for early detection and targeted intervention in hip joint disease.

## 1. Introduction

The hip joint is one of the most biomechanically stable but also the most stressed joints of the human body, being essential for locomotor biomechanics and for maintaining the quality of life in the elderly and active people. Through its complex structure—made up of bone, cartilaginous, ligamentous, and capsular elements—the hip ensures not only mobility but also resistance to constant mechanical overload. However, this complexity also makes it vulnerable to degenerative processes, which can evolve silently and irreversibly, seriously affecting joint biomechanics. Over the past few decades, degenerative hip joint disease has become one of the most common musculoskeletal conditions, with a significant social and economic impact, especially in the context of increasing life expectancy among the population [1]. At the center of this pathological process are not only the articular cartilage but also the periarticular structures, especially the joint capsule. This, although frequently overlooked in clinical practice, plays an essential role in joint biomechanical homeostasis, as well as in the management of the chronic inflammatory response that accompanies joint degradation [2,3,4,5].

The hip joint capsule is a complex fibrous structure, with a collagenous and vascular microarchitecture adapted to both mechanical stresses and biochemical influences of the synovial microenvironment. During the degenerative process, it undergoes significant qualitative, quantitative, and organizational changes, reflecting intense cellular activity, tissue remodeling, and alterations in the microstructure of the extracellular matrix and blood capillaries [6,7,8]. However, in the specialized literature, the capsule has been given relatively little attention compared to other joint components, such as cartilage or subchondral bone [9,10]. Thus, important gaps persist in the understanding of the pathogenic mechanisms that directly involve the joint capsule and how these changes contribute to the evolution and symptomatology of the degenerative disease [11,12,13].

In recent years, technological advances in the fields of scanning electron microscopy (SEM), high-resolution computed tomography (microCT), and immunohistochemistry have allowed for a more detailed exploration of morphofunctional changes in joint structures [14]. These techniques provide complementary insights into tissue architecture, the distribution of matrix elements, and the expression of cellular markers involved in inflammation, fibrosis, angiogenesis, and tissue remodeling [15,16]. The integrated application of these methods in the study of the joint capsule can provide valuable data on the microscopic evolution of the degenerative process and on potential therapeutic or predictive targets [17].

On the other hand, recognizing degenerative changes in the joint capsule is essential not only from a morphological perspective but also from a clinical one [18,19]. Fibrotic remodeling, inflammatory infiltration, and vascular alterations within the capsule contribute to joint pain, mobility restriction, and reduced surgical efficacy, particularly in procedures like total hip arthroplasty [20,21]. Understanding these processes may guide early therapeutic strategies and improve patient selection for orthopedic interventions [17,22].

**Figure 1:**
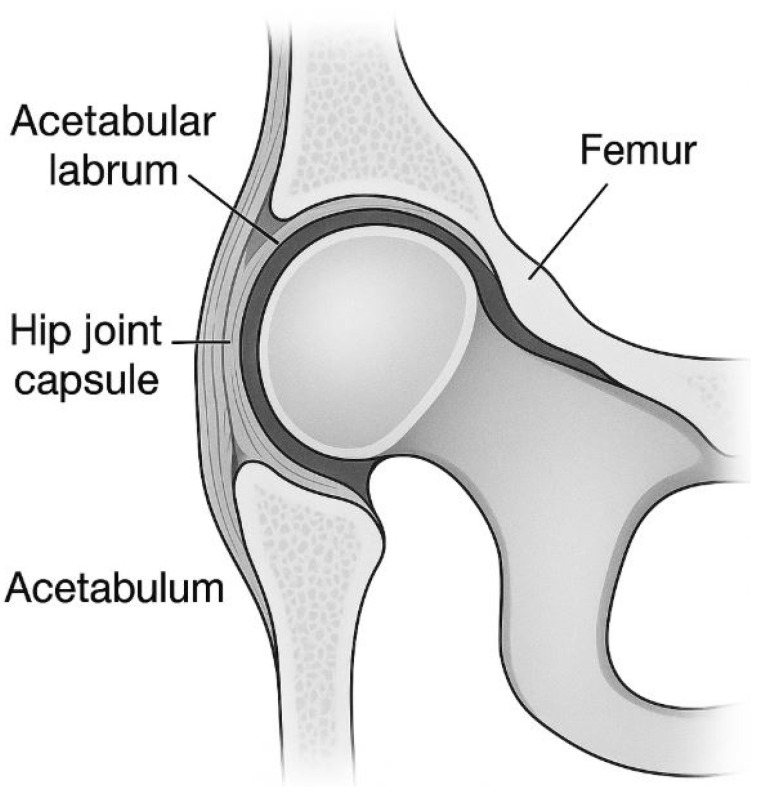
Schematic representation of the hip joint capsule and acetabular labrum anatomy in an oblique coronal section. The illustration highlights the anterosuperior quadrant of the acetabulum, showing the spatial relationship between the fibrous joint capsule and the fibrocartilaginous labrum. This orientation was chosen to emphasize the typical area affected by early degenerative changes, relevant for both arthroscopic access and pathological interpretation.

Histopathologically, the degenerative capsule exhibits a broad spectrum of changes, from fibrotic thickening and collagen network reorganization to altered expression of matrix and cellular proteins. Immunohistochemistry highlights relevant markers, while SEM enables ultrastructural analysis of collagen fiber orientation, and microCT offers insight into the bone–capsule interface [23]. A schematic representation of the joint capsule and acetabular labrum anatomy is provided in Figure 1 to support anatomical understanding.

Closely related to these capsular changes, the acetabular labrum—an important fibrocartilaginous stabilizer—undergoes similar degenerative transformations [24]. Although its central area is relatively avascular, the peripheral region is vulnerable to neovascularization, ischemia, and chronic inflammation. The acetabular labrum shares with the capsule a pathological microenvironment marked by cytokine activation, matrix remodeling, and loss of biomechanical integrity, thus perpetuating a degenerative feedback loop [25].

Both structures arise from specialized mesenchymal tissues and are anatomically and functionally interconnected, with degeneration in one likely impacting the other [26]. Therefore, a simultaneous investigation of both is warranted to understand local and systemic factors influencing joint degeneration. Shared mechanisms—microvascular remodeling, low-grade inflammation, and fibrotic reorganization—appear to drive the progressive decline in structural integrity [27,28,29].

The severity of hip osteoarthritis in our study was classified according to the Kellgren–Lawrence system [30].

The aim of this study is to characterize the histological, immunohistochemical, microstructural (microCT), and ultrastructural (SEM) changes affecting the joint capsule and acetabular labrum in patients with degenerative pathology and to establish potential correlations between these alterations and disease severity.

## 2. Materials and Methods

### 2.1. Study Design

This study includes two groups of patients, from whom tissue samples were collected from the hip joint capsule and the acetabular labrum or articular. The pieces were collected intraoperatively, following hip joint prosthesis interventions, or from formalized cadavers. Tissue samples were taken, mainly, from the chondrolabral junction.

Sample size justification: The sample size was determined based on the exploratory and descriptive nature of the study, which focused on the morphological, immunohistochemical, and ultrastructural characteristics of periarticular hip tissues. A total of 26 cases were included (15 controls and 11 pathological), reflecting the availability of high-quality tissue specimens and the feasibility of performing advanced analyses such as SEM and IHC. This sample size is consistent with prior published studies that utilized similar group sizes for semi-quantitative scoring and correlation analysis in orthopedic and histological research [31,32]. Additionally, commonly accepted methodological recommendations for exploratory studies suggest that sample sizes of 10–15 cases per group are adequate for identifying significant expression patterns and ultrastructural differences, particularly when strict inclusion/exclusion criteria and matched anatomical sites are employed to reduce variability. The number of cases included was based on strict inclusion and exclusion criteria, availability of well-preserved tissue, and feasibility of advanced analyses (including SEM and IHC).

### 2.2. Participants

The first group is a control group consisting of 15 patients without degenerative hip pathology. Of these, 11 were operated on for post-traumatic hip prosthesis, and 4 were formalized cadaveric pieces. The latter were collected from male cadavers aged under 40 years.

Of the 11 operated patients, 5 were women and 6 were men, aged between 27 and 42 years.

The second study group consisted of 11 patients diagnosed with an advanced form of degenerative pathology of the hip joint. They underwent a total hip joint prosthesis operation. The age of these patients ranged between 68 and 79 years. A total of 8 were men and 3 were women. The operated patients in both groups belong to the Orthopedics and Traumatology Clinic of the “St. Spiridon” Emergency Hospital in Iași, from where tissue fragments were taken from the capsule and acetabular labrum of the coxofemoral joint. The tissue samples in this study group were collected during surgical procedures that structurally and functionally restored the hip joint. These patients underwent surgical interventions for total joint prosthesis through the direct lateral Hardinge approach.

For the study of the articular labrum, we used pathological tissue samples collected from 7 of the 11 operated patients. They underwent total hip arthroplasty, and the control group was represented by the 4 cadaveric specimens mentioned.

The inclusion criteria in these study groups consisted of the recommendation of the surgical approach of the hip joint and femoral head prosthesis.

The common exclusion criteria for subjects in the two study groups were refusal of surgery; uncooperative patients or relatives; patients or relatives who did not give their consent to participate in this study; patients who could not undergo surgery due to associated pathology; presence of a congenital bone malformation of the spine and/or lower limbs; and pathologies affecting the osteoarticular system (such as severe scoliosis, hip dysplasia, advanced knee arthrosis, or leg length discrepancy) that could significantly alter postural balance and induce compensatory deformities of the lower limbs or spinal alignment.

The exclusion criteria specific to the control group were age over 50 years, presence of degenerative pathology of the hip joint (evidenced by clinical history or post-mortem imaging), and the presence of peripheral obliterative arteriopathy (assessed by macroscopic vascular inspection during dissection and confirmed by medical records or autopsy findings when available).

The exclusion criteria for the second study group (patients with advanced hip degeneration) were absence of degenerative pathology of the hip joint (as confirmed by pre-operative imaging and intraoperative assessment) and presence of degenerative lesions attributable to non-senescent causes (such as post-traumatic or secondary osteoarthritis), which were excluded based on patient history, surgical findings, and histopathological confirmation.

All patients in the second study group suffered from a cardiovascular disease: 10 were diagnosed with arterial hypertension, 8 with chronic obliterating arteriopathy, 4 with stroke with minor sequelae, and 6 with coronary artery disease requiring vascular prosthesis.

**Figure 2:**
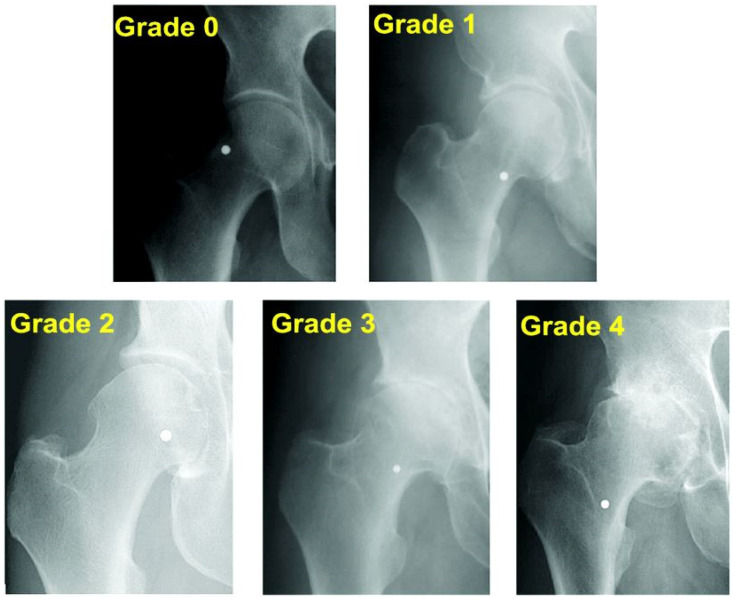
Representative anteroposterior pelvic radiographs illustrating the Kellgren–Lawrence grading system for hip osteoarthritis (Grades 0 to 4). Grade 0: Normal joint with preserved joint space and no osteophytes; Grade 1: Doubtful joint space narrowing and minimal osteophytic lipping; Grade 2: Definite osteophytes and possible joint space narrowing; Grade 3: Moderate osteophytes, definite joint space narrowing, and beginning subchondral sclerosis; Grade 4: Large osteophytes, severe joint space narrowing, pronounced subchondral sclerosis, and femoral head deformity. These radiographs were used as internal reference standards for classification in the pathological group.

The diagnosis and grading of coxarthrosis were established using the Kellgren and Lawrence classification system, based on preoperative standard anteroposterior pelvic radiographs. Each patient was assigned a score from 0 to 4 according to radiographic features such as joint space narrowing, osteophyte formation, subchondral sclerosis, and femoral head deformity. Only patients with grade 3 or 4 coxarthrosis were included in the pathological group. An illustrative figure (Figure 2) has been added to visually summarize the Kellgren-Lawrence scoring criteria [33]. Figure 2 includes anonymized representative radiographs corresponding to each KL score, used as internal reference standards during radiological classification.

### 2.3. Tissue Sample Collection

To reduce variability due to potential confounding factors, we selected pathological and control samples with comparable age ranges and sex distribution, as detailed in Supplementary Table S1. All specimens were obtained from similar anatomical locations of the hip joint to control for loading-related differences in tissue remodeling. However, we acknowledge the limitation inherent to using cadaveric material, which prevents complete demographic and biomechanical matching.

### 2.4. Histological and Immunohistochemical Analysis

Tissue specimens harvested from the joint capsule and acetabular labrum were fixed in 10% neutral buffered formalin, dehydrated in graded alcohols, cleared in xylene, and embedded in paraffin wax. From these blocks, 4–5 µm sections were obtained and mounted on poly-L-lysine-coated slides.

Histological examination was performed using Hematoxylin and Eosin (H&E) and Masson’s Trichrome (Van Gieson) staining for general morphological assessment of connective architecture and fibrosis.

For immunohistochemistry (IHC), the sections were subjected to deparaffinization, rehydration, and heat-induced epitope retrieval (HIER) in citrate buffer (pH 6.0) for 20 min. Endogenous peroxidase activity was blocked with 3% hydrogen peroxide for 10 min at room temperature. Non-specific binding was minimized with blocking buffer (Table 1).

The sections were incubated overnight with primary antibodies in a humidified chamber at 4 °C. Detection was performed using a biotin-streptavidin HRP/DAB system, with HRP-conjugated goat anti-polyvalent secondary antibodies. Chromogenic development was achieved with DAB (3,3′-diaminobenzidine), followed by counterstaining with Mayer’s hematoxylin.

Positive internal controls (normal tissue elements such as blood vessels, connective matrix, or chondrocytes) were included on each slide. Negative controls were processed in parallel with omission of the primary antibody.

The staining intensity and the number of positive cells were semi-quantitatively assessed by two independent observers using a standardized 4-point scoring system, written as follows:

− 0 = negative;
− 1 = weak/low expression;
− 2 = moderate expression;
− 3 = strong/high expression.

Each marker was evaluated separately in both capsule and labral tissues, with scores assigned accordingly. Particular attention was given to nuclear markers (Ki67, SOX9, ERG) and to macrophage and vascular markers (CD68, CD31), along with the expression pattern of Lubricin along surface and pericellular regions. We used anti-ERG and anti-Lubricin antibodies to identify the expression of ERG (a nuclear transcription factor associated with endothelial cells) and Lubricin (a mucinous glycoprotein involved in joint lubrication), respectively.

Ki67 showed an irregular distribution, with strong expression in the cellular components of the Haversian system, particularly within the deeper layers of cortical bone adjacent to the medullary canal.

Photomicrographs were acquired using a calibrated camera mounted on a light microscope (magnifications: 10×, 20×, and 40×), ensuring consistent exposure and resolution across samples.

The intensity and extent of immunohistochemical staining were semi-quantitatively evaluated using a standardized 4-point scale, based on both the percentage of positive cells and the staining intensity, as follows:

− Score 0: No detectable staining or <5% positive cells, regardless of intensity (negative).
− Score 1: Weak staining intensity and/or 5–25% positive cells (low expression).
− Score 2: Moderate intensity and/or 26–50% positive cells (moderate expression).
− Score 3: Strong staining intensity and/or >50% positive cells (high expression).

The scoring was applied to each marker in both the capsule and acetabular labrum tissues, assessing specific cellular compartments (nuclear, cytoplasmic, or membranous) depending on the marker’s localization. Two independent observers, blinded to the clinical group, performed the evaluations to minimize subjective bias.

### 2.5. Surface Electron Microscopy (SEM) Analysis

The ultrastructural study was performed on the same group of patients included in the histological and immunohistochemical studies. Samples collected from the coxofemoral joint of patients who underwent surgical interventions for surgical or anatomical femoral neck fractures, as well as for joint prosthesis, were fixed with formaldehyde and embedded in paraffin wax and from donations from the “Ion Iancu” Institute of Anatomy of our University.

To obtain high-quality images, the high-vacuum working mode of the SEM was employed. Biological samples were fixed in 2.5% glutaraldehyde buffered with 0.1 M sodium cacodylate (pH 7.4), post-fixed in 1% osmium tetroxide, and then dehydrated through a graded ethanol series (30%, 50%, 70%, 90%, and 100%, 10 min each step). After dehydration, the samples were dried at a critical point using carbon dioxide (CO_2_) and subsequently mounted on aluminum stubs. A thin layer of gold (7 nm thickness) was sputter-coated on the surface to ensure electrical conductivity during electron beam scanning.

A state-of-the-art scanning electron microscope, the VegaTescan LMH II, was used. Technical features: 100,000× magnification, secondary electron (SE) detector, high vacuum, tungsten filament, 7-sample carousel (standard dimensions 10 × 10 × 45 mm), and VegaTescan software, version 35.0.0.

For the detailed analysis of the microvascular architecture and articular surfaces in degenerative hip pathology, SEM was used, with a special technical adaptation applicable to histological sections already stained conventionally with hematoxylin-eosin (HE) [34,35]. This approach allowed the valorization of the existing operative biological material, providing a three-dimensional and ultrastructural perspective complementary to the classical morphological investigation.

Tissue samples were initially processed using standard histopathological techniques: fixation in 10% buffered formalin for 24–48 h, dehydration in an ascending alcohol series (70%, 80%, 90%, 96%, and 100%), clarification in xylene, and paraffin embedding. Sections of 4–5 μm thickness were cut, stained with hematoxylin–eosin (H&E), and mounted using synthetic resin-based medium (e.g., DPX). For SEM analysis, selected H&E-stained slides were subjected to a specialized de-mounting protocol using xylene and alcohol gradients to dissolve the mounting medium and gently detach the coverslip without damaging the stained section. No blocking solution was required during SEM preparation, as the protocol did not involve immunohistochemical staining or antibody incubation. This approach allowed the retrieval of intact tissue architecture, offering additional ultrastructural contrast derived from H&E staining. Retrieved sections were rehydrated, post-fixed in 1% osmium tetroxide, dehydrated again in ethanol, and prepared for SEM following standard sputter-coating procedures.

After disassembly, the sections were allowed to dry completely in a controlled environment at room temperature, then they were placed on appropriate metal supports (stubs) for SEM. To ensure the conductivity necessary for examination in vacuum, the surface of the samples was covered with an ultrafine layer of gold (gold-sputtering), with a thickness of approximately 5–10 nm, using a sputter coater device in an argon atmosphere. This step was essential to prevent the accumulation of electrostatic charge during electronic analysis.

SEM examination was performed under medium vacuum conditions, with an accelerating voltage between 5 and 15 kV, using secondary electron detection modes for surface relief and backscattered electrons for compositional contrast. This method allowed the exploration of fine details of the extracellular matrix, trabecular bone structure, vascular organization, and possible changes in mineralization or cartilage degeneration depending on the pathological stage [36].

### 2.6. Statistical Analysis

From a statistical perspective, the primary objective of this study was to evaluate correlations between the results obtained from different investigation techniques. In particular, we aimed to assess the association between the semi-quantitative immunohistochemical (IHC) expression scores and the degree of morphological degradation observed on scanning electron microscopy (SEM). For SEM, a 4-point scoring system (0–3) was developed to evaluate collagen fiber disorganization and surface integrity of the joint capsule and acetabular labrum:

− Score 0—organized, parallel collagen fibers with intact surface topography;
− Score 1—mild disruption with initial signs of fiber fragmentation or waviness;
− Score 2—moderate disorganization, loss of parallel architecture, irregular surfaces;
− Score 3—severe disintegration, collapsed or amorphous fiber structures, highly irregular topography.

These SEM scores were then statistically correlated with the IHC expression scores (described in Section 3.2.2), using Pearson’s and Spearman’s correlation coefficients, depending on distribution normality.

To demonstrate this relationship, appropriate statistical tests were applied, including Pearson’s correlation coefficient (for normally distributed data) or Spearman’s rank correlation coefficient (for ordinal data), both of which quantify the strength of association between the two sets of variables. A Spearman correlation coefficient (ρ) greater than 0.6 was interpreted as a strong positive association, in accordance with widely accepted statistical interpretation scales. For each correlation, 95% confidence intervals were computed to enhance the robustness of interpretation.

A high coefficient value (e.g., ρ > 0.6) would indicate a direct, statistically significant correlation between structural damage and molecular expression, supporting the hypothesis that tissue deterioration is accompanied by enhanced immunohistochemical reactivity. A significant positive correlation (e.g., ρ > 0.6, *p* < 0.05) confirms that as tissue degradation progresses, pathological molecular expression intensifies.

Thus, the statistical findings validate the existence of a consistent relationship between the morphological degree of tissue impairment and its biochemical profile, reinforcing the relevance of both assessment methods in the evaluation of degenerative diseases.

### 2.7. Ethical Considerations

The present study followed the principles outlined in the Declaration of Helsinki. Written informed consent was obtained from each participant who underwent investigation. The ethics committee approval of ‘Grigore T. Popa’ University of Medicine and Pharmacy Iasi number 284, dated 5 March 2023, is attached to this manuscript.

## 3. Results

### 3.1. Results of the IHC Study

#### 3.1.1. Results of the IHC Study on Human Hip Joint Capsule

**Figure 3:**
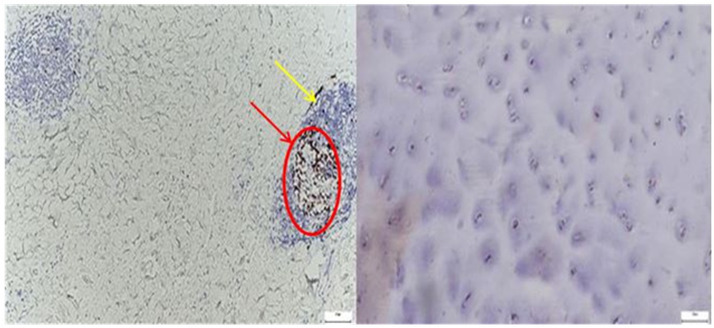
**Left**—Immunohistochemistry for the cell proliferation marker Ki-67 in the hip joint capsule of a patient with degenerative joint disease. Perivascular lymphoid aggregates are seen, with a significant number of positively labeled nuclei (dark brown) in the germinal center of the follicles, indicating localized proliferative activity (red arrow and circle). The underlying tissue shows a reduced cell density and a disorganized collagen network, suggestive of chronic fibrous remodeling (yellow arrow). Magnification: 20×; scale: 50 µm; **Right**—Immunohistochemistry for Ki-67 in the control group. A homogeneous histological appearance is observed, with intact collagen organization and a reduced cell density, typical of healthy capsular tissue. Cell proliferation is minimal, being visible only by the rare presence of a few positively labeled nuclei (brown) in the stroma, without the formation of lymphoid aggregates or follicular proliferative activity. Magnification: 20×; scale: 50 µm.

*Ki67* immunolabeling highlighted a pronounced proliferative response within the pathological capsule, with dense clusters of positive nuclei, particularly in areas of lymphoid aggregation, while in the normal capsule, Ki67 expression was minimal and limited to isolated stromal cells, reflecting low basal cellular turnover (Figure 3).

**Figure 4:**
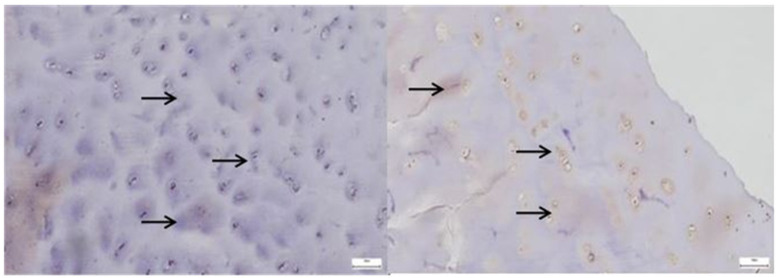
**Left**—Immunohistochemistry for the CD68 marker in the hip joint capsule, taken from a patient with advanced degenerative pathology. Numerous positive cells (dark brown) diffusely dispersed in the stroma are highlighted (black arrows), with perivascular-subsynovial accumulations, corresponding to the infiltrate of activated macrophages. The presence of a small, poorly marked lymphoid aggregate suggests a nonspecific chronic inflammatory reaction. The appearance is suggestive of the involvement of monocyte/microphage components in tissue remodeling and the perpetuation of local chronic inflammation. Magnification: 20×; scale: 50 µm; **Right**—Immunohistochemistry for CD68 in normal hip joint capsule. A sparse distribution of positive cells (dark brown), with a predominantly perivascular location; resident population of tissue macrophages. The absence of diffuse infiltrates or lymphoid organization indicates a non-inflammatory tissue microenvironment, typical of a joint without active pathology. Magnification: 20×; scale: 50 µm.

*CD68*-positive macrophages were markedly more frequent in the pathological hip capsule, displaying an uneven, clustered distribution suggestive of local inflammatory activation, while in the normal capsule, *CD68* expression was sparse and predominantly perivascular, indicating a basal immune surveillance state (Figure 4).

**Figure 5:**
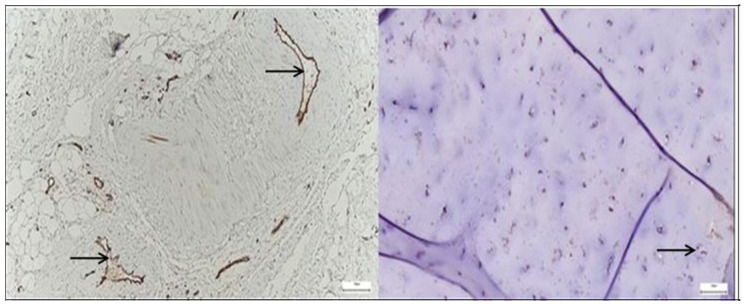
**Left**—Immunohistochemistry for CD31 in the hip joint capsule, taken from a patient with degenerative pathology. An increased density of positive endothelial cells (dark brown) is highlighted, organized as mature vascular structures and neosynthesized capillaries, diffusely distributed in the stroma (black arrows). The appearance reflects an active process of neovascularization and microvascular remodeling, characteristic of chronic inflammation and progressive tissue degradation in degenerative arthropathy. Magnification: 20×; scale: 50 µm; **Right**—Immunohistochemistry for CD31 in a normal hip joint capsule. The presence of small, sparse vascular structures with weakly positive endothelial cells (brown), distributed peripherally and perivascularly, corresponding to a physiological capillary network, is observed. No areas of neovascularization or endothelial proliferation are evident, an appearance characteristic of capsular tissue without inflammation or active remodeling. Magnification: 20×; scale: 50 µm.

*CD31* immunostaining revealed an increased and irregular endothelial signal in the pathological hip joint capsule, with clustered and elongated vascular profiles, whereas the normal capsule exhibited a sparse, evenly distributed expression limited to well-defined microvascular structures (Figure 5).

**Figure 6:**
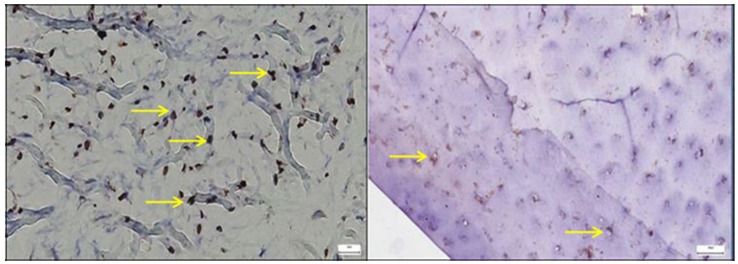
**Left**—In the pathological hip joint capsule, SOX9-positive nuclei appear more numerous and irregularly distributed within a disorganized fibrous matrix (yellow arrows), suggesting a reactive or compensatory upregulation of this transcription factor in response to structural and functional tissue alterations; **Right**—SOX9-positive nuclei are evenly distributed throughout the fibrous layer of the normal hip joint capsule, with stronger expression near fibrocartilaginous regions, reflecting its physiological role in maintaining matrix integrity and supporting joint tissue homeostasis, 40× bilateral.

In the pathological hip joint capsule, *SOX9* immunoreactivity was increased, with numerous positively stained nuclei dispersed throughout a disorganized connective matrix, contrasting with the more uniform and sparse expression observed in normal tissue (Figure 6). *SOX9* expression in the normal hip joint capsule appears as a well-preserved architecture, with the fibrous connective tissue matrix of the capsule clearly delineated. The staining pattern is nuclear, consistent with the known localization of SOX9, a transcription factor critically involved in chondrogenesis and extracellular matrix regulation. The image shows discrete nuclear positivity for SOX9 within the fibrous layer of the capsule, with evenly dispersed stained nuclei and no cytoplasmic signal. The expression pattern reflects baseline transcriptional activity associated with matrix homeostasis and fibrocartilaginous cell maintenance.

**Figure 7:**
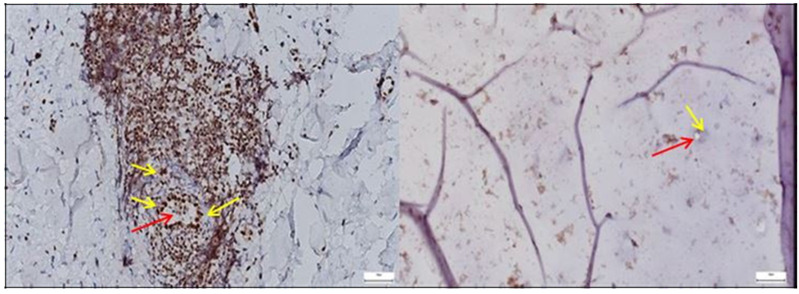
**Left**—In the pathological hip joint capsule, ERG immunostaining (yellow arrows) revealed a heterogeneous pattern, with scattered nuclear positivity primarily confined to endothelial cells (red arrows) lining irregular microvascular structures, indicating altered angiogenic activity and potential vascular remodeling in the affected tissue; **Right**—In the normal hip joint capsule, ERG immunostaining showed discrete nuclear positivity restricted to endothelial cells of well-organized microvessels, consistent with physiological vascular architecture and baseline endothelial integrity, 25× bilateral.

Compared to the normal hip joint capsule, where *ERG* expression was confined to orderly microvascular endothelial nuclei, the pathological capsule displayed a more irregular and diffuse *ERG* staining pattern, reflecting disrupted vascular architecture and potential endothelial activation or remodeling (Figure 7).

**Figure 8:**
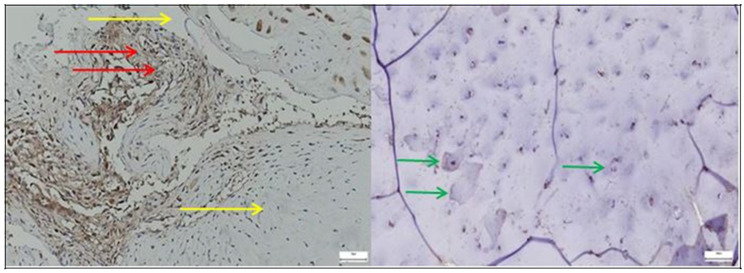
**Left**—In the pathological hip joint capsule, Lubricin immunostaining revealed patchy and diminished expression (yellow arrows), with weak, or absent signal in areas of matrix disorganization (red arrows), suggesting impaired boundary lubrication and possible involvement in degenerative tissue changes. **Right**—In the normal hip joint capsule, Lubricin expression (green arrows) was clearly detected along the surface layers and within pericellular zones, with a continuous and well-defined immunostaining pattern, supporting its physiological role in joint lubrication and protection against mechanical stress, 40× bilateral.

Regarding the aspect of the normal capsule, where *Lubricin* showed a continuous and well-demarcated expression pattern along surface and pericellular regions, the pathological hip joint capsule exhibited a fragmented and reduced staining signal, suggesting disrupted lubrication mechanisms and matrix integrity loss associated with degenerative changes (Figure 8).

#### 3.1.2. Results of the IHC Study on Human Hip Joint Acetabular Labrum

**Figure 9:**
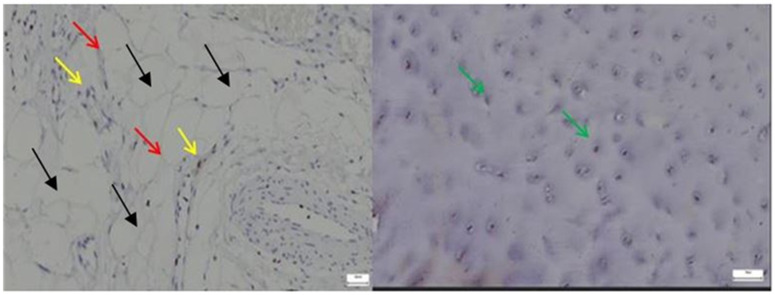
**Left**—Ki67 immunohistochemical staining in the pathological acetabular labrum of the hip joint. The image shows scattered Ki67-positive nuclei (yellow arrows) within the fibrous and perivascular areas of the labral tissue, indicating localized proliferative activity. Increased nuclear labeling is observed particularly around vascular structures (red arrows) and within stromal regions, suggestive of ongoing remodeling or reparative responses associated with degenerative or inflammatory changes; black arrows mark the presence of adipose tissue. **Right**—Ki67 immunostaining in normal labral tissue. The image shows rare Ki67-positive nuclei within the fibrous matrix of the acetabular labrum, with most cells displaying no proliferative activity. The low density and scattered distribution of labeled nuclei are consistent with a quiescent cellular profile typical of healthy labral tissue under physiological conditions, 50× bilateral.

*Ki67* expression was markedly elevated in the pathological acetabular labrum, with frequent nuclear labeling observed in stromal and perivascular regions, whereas the normal labral tissue exhibited only sparse and isolated *Ki67*-positive nuclei, reflecting minimal baseline proliferative activity (Figure 9).

The image on the left, corresponding to the pathological hip joint capsule, reveals multiple large, optically clear, well-demarcated vacuoles, consistent with the histological appearance of mature adipocytes. These structures display peripheral nuclei, a hallmark feature of white adipose tissue. The yellow and red arrows delineate these regions, which appear to infiltrate or replace the native connective matrix.

By contrast, the control image on the right lacks such features. It demonstrates a homogeneous fibrous architecture, with evenly distributed chondrocyte-like cells (highlighted by green arrows) and no evidence of fatty infiltration or stromal disarray.

The emergence of adipose tissue within pathological capsules may suggest a process of adipogenic metaplasia in response to chronic inflammation or progressive structural deterioration, or a phenomenon of stromal fatty infiltration, commonly associated with advanced degenerative changes or accelerated aging of the capsular tissue.

**Figure 10:**
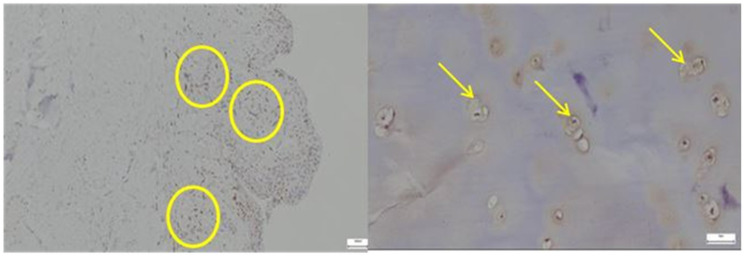
**Left**—CD68 immunostaining in pathological labral tissue. Numerous CD68-positive macrophages are evident (yellow circle), displaying a clustered and uneven distribution within the fibrous matrix and perivascular zones. This increased immunoreactivity reflects a prominent inflammatory response and suggests active monocyte-macrophage involvement in the pathological remodeling of the acetabular labrum; **Right**—CD68 immunostaining in normal labral tissue (yellow arrows), 40× bilateral.

CD68 expression was markedly elevated in the pathological acetabular labrum, with numerous macrophages aggregated within the stroma and perivascular regions, in contrast to the normal acetabular labrum, where only sparse CD68-positive cells were observed, reflecting minimal baseline immune activity (Figure 10).

Only occasional CD68-positive macrophages are visible within the stromal compartment, primarily localized in perivascular areas. CD68-positive cells were sparsely distributed, mainly in the perivascular areas of the connective tissue, without forming prominent clusters. This aspect corresponds to a normal histological appearance in labral tissue without visible signs of inflammation.

**Figure 11:**
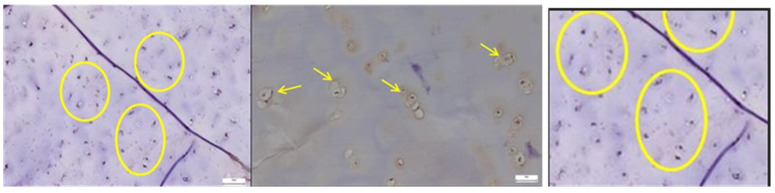
**Left**—CD31 immunostaining in pathological labral tissue. The image reveals prominent CD31 expression in endothelial cells lining irregular and often enlarged microvascular profiles (yellow circle). The increased density and disorganized arrangement of CD31-positive vessels suggest active neoangiogenesis and vascular remodeling associated with pathological tissue changes; **Midddle**—CD31 immunostaining in normal labral tissue. Sparse CD31-positive endothelial cells are identified within well-formed microvascular structures (yellow arrows). The orderly distribution and low vessel density are consistent with the physiological vascular architecture of healthy labral tissue, lacking signs of angiogenic activation, 40× bilateral. The image on the **right** is a zoomed detail from the left panel, highlighting cell clusters and disrupted fiber arrangement in the degenerative capsule tissue (yellow circles).

In the pathological acetabular labrum, CD31 immunostaining revealed a dense and disorganized network of endothelial cells lining enlarged or irregular microvessels, indicative of active neovascularization, whereas in the normal acetabular labrum, CD31 expression was limited to sparse, well-organized vascular profiles, reflecting a stable physiological microcirculation (Figure 11).

**Figure 12:**
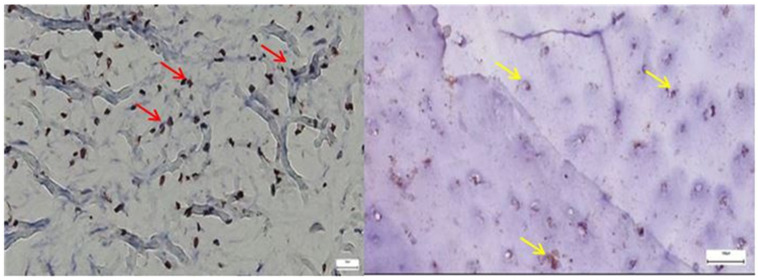
**Left**—SOX9 immunostaining in pathological labral tissue (red arrows). The image displays an increased number of SOX9-positive nuclei, irregularly distributed within the fibrous matrix. The heightened and disorganized expression pattern suggests altered transcriptional regulation, possibly reflecting reactive or reparative responses to degenerative changes within the acetabular labrum; **Right**—SOX9 immunostaining in normal labral tissue (yellow arrows). Scattered SOX9-positive nuclei are observed within the fibrous component of the acetabular labrum, showing a low and uniform nuclear staining pattern. This limited expression is consistent with the physiological role of SOX9 in maintaining matrix integrity under non-pathological conditions, 25× bilateral.

*SOX9* expression was markedly increased and irregularly distributed in the pathological acetabular labrum, suggesting transcriptional activation related to matrix remodeling, whereas in the normal acetabular labrum, *SOX9*-positive nuclei were sparse and uniformly scattered, reflecting baseline homeostatic activity (Figure 12).

**Figure 13:**
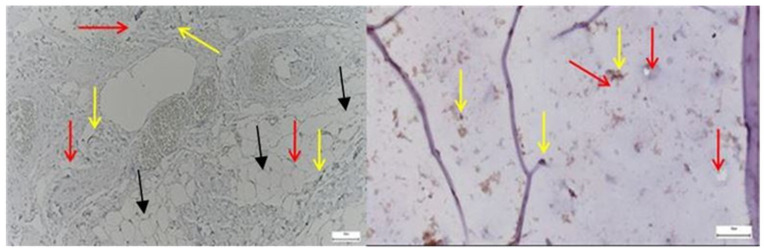
**Left**—ERG immunostaining in pathological labral tissue. ERG-positive nuclei are prominently detected in endothelial cells lining irregular and densely packed microvessels (red arrows). The increased staining intensity and vascular complexity suggest endothelial activation and pathological neovascularization, consistent with vascular remodeling processes in degenerative labral tissue (yellow arrows); black arrows mark the presence of adipose tissue, with similar impact as in Figure 9; **Right**—ERG immunostaining in normal labral tissue. Sparse ERG-positive nuclei are observed within well-formed endothelial linings of small, regularly distributed microvessels. The low vascular density and orderly architecture are consistent with a quiescent endothelial phenotype typical of non-pathological labral tissue, 40× bilateral.

*ERG* immunostaining revealed a marked increase in endothelial labeling within the pathological acetabular labrum, characterized by irregular, densely packed microvessels suggestive of neoangiogenesis, while in the normal acetabular labrum, ERG expression was limited to sparse, well-structured vascular profiles, reflecting stable microvascular architecture (Figure 13).

**Figure 14:**
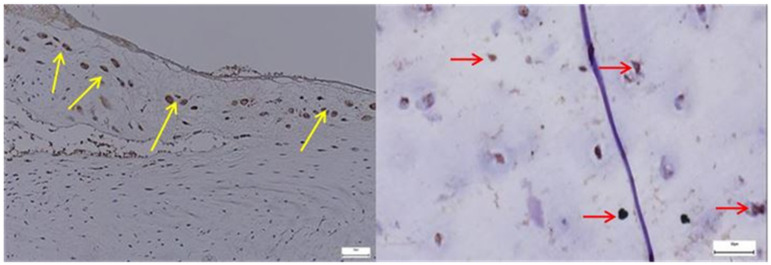
**Left**—Lubricin immunostaining in pathological labral tissue. The image shows a discontinuous and diminished Lubricin signal along the surface and pericellular regions of the acetabular labrum (yellow arrows). Fragmented and patchy expression patterns suggest compromised boundary lubrication and potential alterations in matrix integrity associated with degenerative changes; **Right**—Lubricin immunostaining in normal labral tissue (red arrows). A continuous and well-defined Lubricin signal is observed along the surface and pericellular regions of the acetabular labrum. This uniform expression pattern reflects intact boundary lubrication and matrix integrity, consistent with physiological homeostasis in healthy tissue, 40× bilateral.

Lubricin expression was continuous and well-preserved in the normal acetabular labrum, outlining surface and pericellular areas, whereas in the pathological acetabular labrum, the signal appeared discontinuous and weakened, suggesting impaired lubrication and structural degradation linked to degenerative changes (Figure 14).

### 3.2. Results of the SEM Study

#### 3.2.1. Results of the SEM Study on Human Hip Joint Capsule

**Figure 15:**
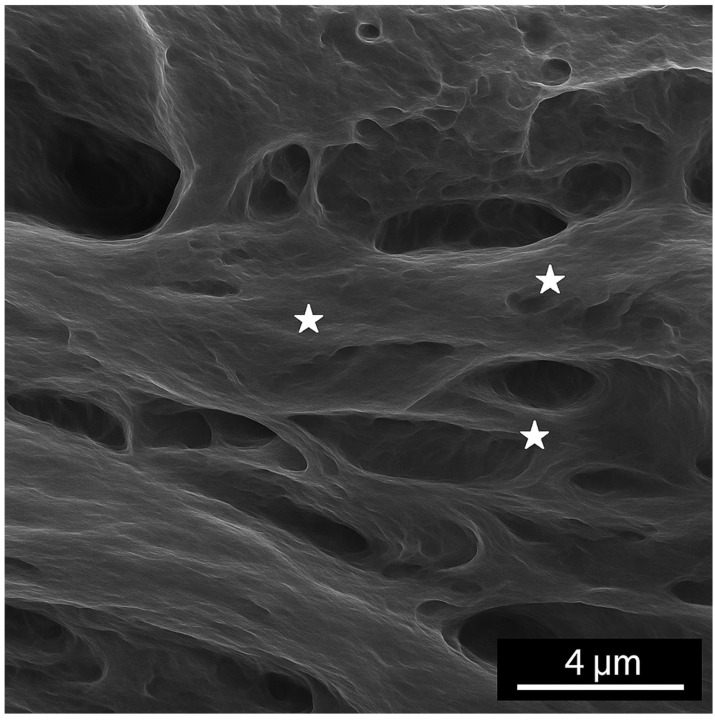
Scanning electron microscopy (SEM) image of the normal hip joint capsule. White stars indicates connective tissue.

**Figure 16:**
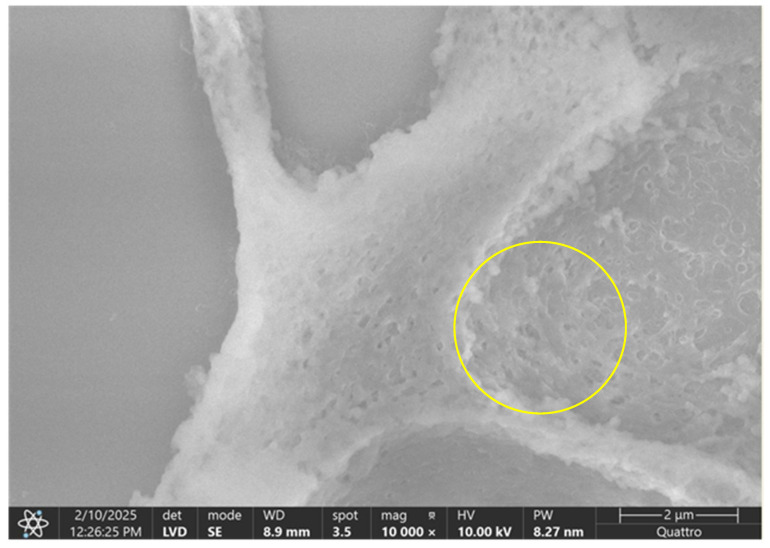
SEM image of the pathological hip joint capsule. The image illustrates a disrupted connective tissue structure, with partial loss of the typical stratified architecture. Collagen fibers appear fragmented and disorganized, lacking the parallel alignment seen in healthy tissue (yellow circle). The surface is irregular, with regions of matrix disintegration, indicating degenerative remodeling. These ultrastructural alterations suggest compromised mechanical integrity and tissue homeostasis.

Compared to the normal hip joint capsule, which exhibited a well-organized, stratified collagen architecture with parallel fiber alignment, the pathological capsule showed marked ultrastructural disorganization, with fragmented collagen bundles, loss of stratification, and irregular surface morphology indicative of degenerative remodeling (Figure 15 and Figure 16).

The image reveals a well-organized, stratified connective tissue architecture, with a dense fibrous layer at the surface and a deeper, less compact zone suggestive of a transitional interface. Collagen fibers appear uniformly arranged, running in parallel bundles, without signs of structural disruption or surface degradation. This ultrastructural organization supports the mechanical resilience and physiological integrity of the normal capsule.

#### 3.2.2. Results of the SEM Study on Human Hip Joint Acetabular Labrum

**Figure 17:**
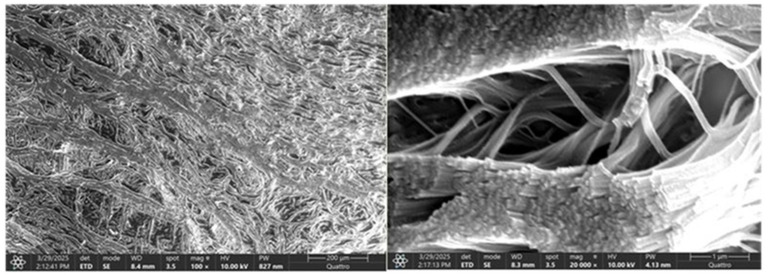
Scanning electron microscopy (SEM) images of normal labral tissue—both panels illustrate the organized ultrastructural architecture of the healthy acetabular labrum. The surface appears smooth and continuous, with well-aligned collagen fibrils arranged in parallel bundles, indicative of mechanical integrity. No signs of surface disruption, fiber fragmentation, or matrix degradation are present, supporting the preservation of native extracellular matrix morphology under physiological conditions.

**Figure 18:**
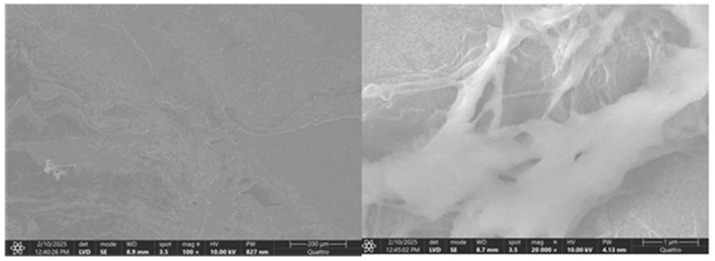
Scanning electron microscopy (SEM) images of pathological labral tissue—the images reveal marked ultrastructural disorganization of the labral matrix. Collagen fibers appear fragmented, irregularly oriented, and poorly compacted, with visible surface discontinuities and porous zones. These morphological alterations suggest matrix degradation and compromised mechanical stability, consistent with degenerative remodeling processes in the pathological acetabular labrum.

SEM analysis revealed a well-organized collagenous architecture in the normal acetabular labrum, with parallel fibrillar alignment and intact surface morphology, while the pathological acetabular labrum exhibited pronounced ultrastructural disruption, including fragmented fibers, disorganized orientation, and surface discontinuities indicative of matrix degeneration (Figure 17 and Figure 18).

### 3.3. Statistical Analisis Results

#### 3.3.1. IHC Results of Statistical Study

The immunohistochemical (IHC) analysis performed on tissue samples from the hip capsule and acetabular labrum revealed distinct expression patterns of key molecular markers involved in inflammation, cellular proliferation, vascularization, and extracellular matrix integrity. Comparative scoring across the pathological and control groups included markers such as Ki67, CD68, CD31, SOX9, ERG, and Lubricin. The pathological group consistently exhibited higher expression scores for markers associated with inflammatory infiltration (CD68), endothelial activation (CD31, ERG), and proliferative activity (Ki67), suggesting a more pronounced tissue remodeling process. Conversely, the control group displayed lower scores across all markers, reflecting a relatively preserved and quiescent tissue state (Table 2 and Table 3).

**Table 2:** Table presenting the immunohistochemical (IHC) scoring results for tissue samples collected from the hip capsule of patients with degenerative pathology (pathological group) and individuals with morphologically normal joints (control group).

Patient ID	Group	Ki67	CD68	CD31	SOX9	ERG	Lubricin
1	Pathological	3	3	3	3	2	1
2	Pathological	3	3	3	3	2	1
3	Pathological	3	2	3	3	2	0
4	Pathological	2	2	3	3	3	0
5	Pathological	3	2	3	3	3	0
6	Pathological	3	2	2	3	2	0
7	Pathological	2	2	3	3	3	1
8	Pathological	3	2	3	3	2	1
9	Pathological	3	3	3	3	3	1
10	Pathological	3	3	2	3	2	1
11	Pathological	3	2	3	2	2	1
12	Pathological	3	3	3	3	3	1
13	Pathological	2	2	3	3	2	1
14	Pathological	3	2	3	3	3	1
15	Pathological	3	2	2	2	3	0
16	Control	1	0	1	0	0	2
17	Control	0	1	1	1	1	3
18	Control	0	0	1	1	1	2
19	Control	1	1	1	1	1	2
20	Control	1	1	0	0	1	2
21	Control	1	1	1	0	1	2
22	Control	1	0	1	0	1	3
23	Control	1	1	1	1	1	2
24	Control	1	1	1	1	1	2
25	Control	1	1	1	1	0	3
26	Control	0	0	0	0	1	2

The scoring was applied per case and per marker, focusing on six molecular markers relevant to inflammation, proliferation, vascularization, chondrogenesis, and lubrication:

- Ki67: Nuclear protein expressed during cell proliferation; high scores indicate increased mitotic activity.
- CD68: Marker of macrophage infiltration; elevated values suggest an active inflammatory response.
- CD31: Endothelial marker indicating vascular density and neovascularization processes.
- SOX9: Transcription factor essential for chondrocyte differentiation and cartilage matrix maintenance.
- ERG: Nuclear transcription factor associated with endothelial integrity and angiogenesis.
- Lubricin: Glycoprotein responsible for joint lubrication; reduced levels are indicative of mechanical stress and cartilage deterioration.

Each marker was scored on a scale from 0 to 3:

- 0—absent or minimal expression,
- 1—low expression,
- 2—moderate expression,
- 3—high expression.

The data allows for comparative profiling between pathological and control tissues, highlighting distinct molecular changes associated with degenerative joint disease.

**Table 3:** Table presenting the immunohistochemical (IHC) scoring results for tissue samples collected from the acetabular labrum of patients with degenerative pathology (pathological group) and individuals with morphologically normal joints (control group).

Patient ID	Group	Ki67	CD68	CD31	SOX9	ERG	Lubricin
1	Pathological	2	3	3	3	3	0
2	Pathological	2	2	2	3	2	1
3	Pathological	3	2	3	3	3	0
4	Pathological	2	3	3	3	3	0
5	Pathological	3	3	2	3	3	0
6	Pathological	2	2	2	2	2	0
7	Pathological	2	3	3	3	3	1
8	Pathological	2	3	2	3	2	1
9	Pathological	2	3	3	3	3	0
10	Pathological	2	2	2	3	3	1
11	Pathological	2	3	3	2	2	1
12	Pathological	2	3	2	3	3	0
13	Pathological	3	3	3	3	3	1
14	Pathological	3	2	3	3	3	1
15	Pathological	2	3	3	3	3	0
16	Control	1	1	1	1	1	2
17	Control	1	1	1	1	1	2
18	Control	1	1	1	1	1	2
19	Control	1	1	1	1	1	2
20	Control	1	1	1	1	1	2
21	Control	1	1	1	1	1	2
22	Control	0	1	0	1	1	3
23	Control	1	0	1	1	1	2
24	Control	0	1	1	1	1	2
25	Control	1	1	1	1	0	2
26	Control	1	1	1	1	1	2

The scoring was applied per case and per marker, focusing on six molecular markers relevant to inflammation, proliferation, vascularization, chondrogenesis, and lubrication:

- Ki67: Nuclear protein expressed during cell proliferation; high scores indicate increased mitotic activity.
- CD68: Marker of macrophage infiltration; elevated values suggest an active inflammatory response.
- CD31: Endothelial marker indicating vascular density and neovascularization processes.
- SOX9: Transcription factor essential for chondrocyte differentiation and cartilage matrix maintenance.
- ERG: Nuclear transcription factor associated with endothelial integrity and angiogenesis.
- Lubricin: Glycoprotein responsible for joint lubrication; reduced levels are indicative of mechanical stress and cartilage deterioration.

Each marker was scored on a scale from 0 to 3:

- 0—absent or minimal expression,
- 1—low expression,
- 2—moderate expression,
- 3—high expression.

The data allows for comparative profiling between pathological and control tissues, highlighting molecular changes associated with degenerative processes in the acetabular labrum.

#### 3.3.2. SEM Results of the Statistical Study

The scanning electron microscopy (SEM) analysis enabled detailed morphological evaluation of bone, cartilage, and capsular tissues obtained from patients with degenerative hip pathology and control subjects with normal joint structure. Scoring was conducted across three key parameters—structural continuity and integrity, collagen presence and fibrillar organization, and vascularization—using a standardized scale from 0 (normal or absent) to 3 (severely altered or extensive). This approach provided a semi-quantitative framework to distinguish pathological changes in microarchitecture, extracellular matrix, and microvascular features between the two groups. The aggregated scores per tissue type and pathological status revealed consistent differences, particularly in the pathological group, which showed more severe disruption of tissue architecture and higher vascularization scores (Table 4A,B).

These are the results of the two separate SEM score tables for cartilage (Section A) and capsular tissue (Section B) obtained from patients with degenerative hip pathology (pathological group) and from individuals with normal joint structures (control group).

Scoring criteria (0–3 points each) are:

- Structural continuity/integrity: 0 = intact structure, 3 = severely affected.
- Presence of collagen/fibrillar organization: 0 = no collagen, 3 = well-organized matrix.
- Vascularization/visible capillaries: 0 = absent, 3 = extensive vascular network.

The total scores reflect cumulative SEM evaluations across multiple samples for each group and structure.

This study assessed degenerative changes in the hip joint by combining ultrastructural analysis via SEM and molecular evaluation through IHC. Analyses were performed separately on two key anatomical structures: the hip capsule and the acetabular labrum. Comparative scoring between the pathological and control groups was conducted using nonparametric statistical methods, and correlations between structural deterioration and molecular expression were evaluated.

##### SEM Score Distribution

In the control group, SEM scores were uniformly zero across all structural categories (*n* = 15). In contrast, the pathological group (*n* = 11) showed significant ultrastructural degradation, with cumulative scores in the capsule of 15 (continuity), 21 (collagen organization), and 16 (vascularization). For the acetabular labrum, similar values were recorded. Mann–Whitney U tests confirmed statistically significant differences for all three parameters between groups (*p* < 0.001 for each comparison).

##### IHC Score Distribution

The IHC analysis focused on six markers: Ki67 (proliferation), CD68 (macrophages), CD31 (endothelium), SOX9 (chondrogenesis), ERG (angiogenesis), and Lubricin (joint lubrication). In the capsule, the pathological group (*n* = 15) showed elevated cumulative scores for Ki67 (41), CD68 (38), CD31 (43), SOX9 (44), ERG (41), and a decreased Lubricin score (14), compared to the control group (*n* = 11) with lower cumulative scores: Ki67 (9), CD68 (7), CD31 (10), SOX9 (9), ERG (10), and significantly higher Lubricin (33). Similar trends were observed in the acetabular labrum: pathological group scores were Ki67 (37), CD68 (43), CD31 (43), SOX9 (43), ERG (43), and Lubricin (9); while the control group recorded Ki67 (11), CD68 (11), CD31 (12), SOX9 (12), ERG (10), and Lubricin (33). Mann–Whitney U test results revealed statistically significant differences for most markers (*p* < 0.05), especially for Ki67, CD68, ERG, and Lubricin.

##### Correlation Between SEM and IHC

Spearman correlation coefficients were calculated to evaluate the relationship between SEM degradation scores and IHC marker expression levels in the pathological group. In the capsule, a moderate-to-strong positive correlation was found (ρ = 0.66, *p* = 0.047), indicating that greater ultrastructural damage was associated with increased molecular reactivity. In the acetabular labrum, the correlation was slightly stronger (ρ = 0.71, *p* = 0.032). These findings confirm the hypothesis that morphological disintegration is paralleled by molecular dysregulation, validating the complementary nature of SEM and IHC in characterizing joint degeneration.

## 4. Discussion

The present study provides an integrated immunohistochemical and ultrastructural analysis of the hip joint capsule and acetabular labrum, comparing normal and pathological specimens. Using a panel of markers (Ki67, CD68, CD31, SOX9, ERG, and Lubricin) alongside SEM, the study elucidates key changes in cell proliferation, immune activity, vascularization, transcriptional regulation, and surface integrity. These findings contribute to the current understanding of degenerative joint processes and their structural-functional consequences.

### 4.1. Proliferative and Inflammatory Markers

The increased Ki67 expression observed in pathological capsule and acetabular labrum samples highlights a localized hyperproliferative response, consistent with tissue remodeling or reparative activity. Similar patterns of proliferation have been reported in degenerative joint conditions, particularly in response to mechanical stress and inflammation [32]. Concomitantly, the upregulation of CD68-positive macrophages in pathological tissues suggests an active inflammatory milieu, which may potentiate matrix degradation and contribute to angiogenesis via cytokine release [37]. This inflammatory activation aligns with prior reports in osteoarthritis and femoroacetabular impingement syndromes, where macrophage infiltration plays a critical role in tissue degeneration [38].

### 4.2. Vascular and Endothelial Changes

CD31 and ERG immunostaining demonstrated marked microvascular remodeling in pathological tissues, with increased vascular density and disorganized architecture. These findings indicate an angiogenic switch likely driven by hypoxic or inflammatory stimuli. Such vascular changes have been shown to exacerbate joint degeneration by promoting infiltration of immune cells and disrupting tissue homeostasis [39,40]. Notably, ERG, a nuclear marker of endothelial integrity, showed irregular expression in pathological conditions, suggesting endothelial activation and dysfunction.

### 4.3. Transcriptional and Matrix-Related Findings

SOX9, a key regulator of chondrogenic and fibrocartilaginous matrix maintenance, exhibited higher and more disorganized expression in pathological samples. This may reflect an attempted reparative response, as SOX9 is typically reactivated during cartilage injury and repair [41]. However, the lack of spatial organization and increased staining intensity point to a dysregulated regenerative process. In contrast, Lubricin, a surface-lubricating glycoprotein critical for joint protection, was significantly reduced and fragmented in pathological tissues. This finding correlates with previous reports demonstrating decreased Lubricin in degenerative joint disease, contributing to increased friction and mechanical damage [42].

The presence of adipose-like vacuolated areas, exclusively in the pathological capsules (Figure 9, left), may reflect stromal remodeling with fatty infiltration—a phenomenon occasionally reported in chronically degenerated fibrous tissues such as tendons or joint capsules [43]. This finding supports the hypothesis of advanced tissue deterioration involving both extracellular matrix disorganization and mesenchymal lineage shift toward adipogenic phenotypes [43].

The comparative IHC scoring highlights a consistent upregulation of inflammatory, proliferative, and angiogenic markers in both the capsule and acetabular labrum of pathological samples. These findings align with the current literature suggesting that chronic degenerative processes in the hip joint are marked by increased macrophage presence (CD68), neovascularization (CD31, ERG), and heightened cellular turnover (Ki67). The reduced expression of Lubricin, particularly in pathological specimens, may indicate a compromised joint lubrication system, contributing to further mechanical stress and matrix degradation. The presence of SOX9, a transcription factor critical in chondrogenesis, in both pathological and control tissues suggests a baseline regenerative attempt, although its consistent levels in the pathological group may reflect a stalled repair mechanism. These immunohistochemical patterns support the notion that synovial and fibrocartilaginous tissues in degenerative hip conditions undergo coordinated, yet potentially maladaptive, molecular remodeling.

### 4.4. Ultrastructural Alterations (SEM)

SEM analysis further corroborated the histological findings. Normal capsules and labra exhibited organized collagen fiber alignment and smooth surface morphology, while pathological samples showed fiber fragmentation, disarray, and surface irregularities. These morphological disruptions correspond with decreased tensile strength and elasticity in degenerated tissues and mirror observations in other synovial joints under mechanical stress or inflammatory insult [44].

The comparative SEM scoring revealed pronounced structural and biochemical remodeling in both bone/cartilage and capsular tissues from patients with degenerative hip pathology. The pathological group exhibited significantly higher scores for structural disintegration, collagen matrix disorganization, and increased vascular presence, indicative of active degenerative processes. In contrast, the control group maintained preserved architecture with minimal vascularization. These findings align with current evidence suggesting that chronic joint degeneration involves matrix degradation, fibrovascular proliferation, and loss of microstructural integrity. The changes observed in the capsule, mirroring those in subchondral structures, underscore its role not only as a passive envelope but also as an active participant in pathological remodeling. SEM scoring thus serves as a valuable tool for correlating morphological damage with functional deterioration in degenerative joint disease.

The statistical outcomes of this study confirm that both the capsule and acetabular labrum undergo profound structural and biochemical changes in the context of hip joint degeneration. SEM revealed a consistent pattern of degradation across the pathological group, with loss of tissue integrity, disorganized collagen networks, and increased microvascular presence. These changes were not observed in the control group, highlighting their specificity for pathological remodeling.

IHC analysis further validated these observations, with significant upregulation of proliferative (Ki67), angiogenic (ERG, CD31), and inflammatory (CD68) markers, coupled with a pronounced downregulation of Lubricin—a key marker of joint lubrication and integrity. These molecular findings underscore the systemic nature of joint degeneration, bridging microscopic breakdown with dynamic cellular responses.

The statistical correlation between SEM and IHC scores strengthens the argument for an integrative evaluation model. In both the capsule and acetabular labrum, higher SEM scores were significantly associated with elevated IHC marker expression, particularly for Ki67, CD68, and ERG. This suggests that morphological degradation and molecular locally adapted inflammatory cascade progress hand-in-hand, offering multiple diagnostic entry points into the disease process. The incorporation of both imaging and immunohistochemical criteria may enhance diagnostic accuracy and provide a more robust framework for future therapeutic targeting.

This pattern is consistent with previous studies that describe similar immunohistochemical and ultrastructural changes in joint-related connective tissues affected by osteoarthritis or age-related degeneration [45,46]. Rather than indicating a true dysregulation, the findings reflect a physiological adaptation to sustained mechanical and metabolic stress, commonly seen in the context of degenerative joint disease.

The findings of our study align with recent evidence reported by Haubruck et al. (2021) [6] and Matta et al. (2025) [24], both of which describe low-grade, compartmentalized inflammation and progressive matrix disorganization in hip and knee osteoarthritis. Similar to our observations, these studies highlight the role of resident macrophages and altered expression of matrix remodeling proteins (e.g., MMPs, Lubricin) in driving tissue adaptation rather than overt pathology. Notably, the parallel ultrastructural changes captured via SEM in our samples confirm the degenerative–inflammatory interface described molecularly in these works, supporting a shared pathogenic model of biomechanically modulated joint degradation.

Our findings provide novel insight into the parallel involvement of the hip joint capsule and acetabular labrum in degenerative pathology, combining immunohistochemical markers with ultrastructural observations. While previous studies have focused primarily on cartilage degradation or labral tears, few have explored the microvascular and inflammatory dynamics of periarticular soft tissues in such detail. By identifying a consistent pattern of macrophage infiltration (CD68), neovascularization (VEGF), and matrix disorganization (Lubricin, SEM), our work expands current understanding beyond structural changes alone. In contrast to earlier reports that treated the capsule and labrum in isolation, our integrative approach reveals a shared degenerative microenvironment, emphasizing the importance of these tissues in disease progression and potentially in surgical outcomes.

### 4.5. Study Limitations

This study is limited by its descriptive nature and relatively small sample size. Quantitative morphometric or digital image analysis was not employed, which could provide further insights into the extent of molecular and structural changes. Additionally, patient variability in terms of age, sex, and underlying pathology may have introduced biological heterogeneity. Future studies should include functional correlation with clinical data and expand on longitudinal observations to better define progression.

The integration of ultrastructural and immunohistochemical analyses in both the hip capsule and acetabular labrum offers valuable translational insights into the pathophysiology of joint degeneration. By demonstrating consistent molecular and structural patterns across these periarticular structures, the study reinforces the notion that the degenerative process is not isolated to articular cartilage or subchondral bone but involves a coordinated breakdown of supportive tissues critical to joint stability and function. From a clinical standpoint, the correlation between capsule and acetabular labrum alterations suggests that biopsies or targeted evaluations of one structure may reflect broader joint pathology. This has important implications for minimally invasive diagnostics, surgical planning (e.g., labral repair vs. capsulotomy), and the development of composite biomarkers for early-stage detection. Moreover, the demonstrated statistical concordance between SEM and IHC parameters enhances the credibility of these tools as complementary diagnostic approaches, particularly in distinguishing inflammatory, proliferative, and matrix-degrading components of disease. The capsule–labrum comparative model proposed here may serve as a conceptual and methodological framework for future multidisciplinary research in degenerative joint disorders.

One of the main limitations of this study is the lack of systematic clinical scoring data (e.g., functional scores or pain levels) that would have enabled direct correlation between histological/immunohistochemical changes and clinical status. While the severity of degenerative changes was assessed radiologically using the Kellgren–Lawrence classification, future studies should incorporate standardized clinical evaluations to explore the functional impact of molecular and structural remodeling in the joint capsule and acetabular labrum.

## 5. Conclusions

The combined immunohistochemical and SEM findings support the hypothesis that degenerative changes in the hip joint capsule and acetabular labrum involve a complex interplay of hyperproliferation, immune activation, angiogenesis, impaired transcriptional regulation, and matrix breakdown. The altered expression patterns of SOX9 and Lubricin may serve as potential biomarkers of early tissue degeneration. These insights open the path for further translational research targeting molecular pathways involved in joint preservation and regeneration and suggest future opportunities for biologically guided interventions in early hip joint disease.

## Figures and Tables

**Table 1:** Primary antibodies used for IHC.

Antibody	Reference	Source	Dilution
Ki67	MM1	Abcam, UK	1:100
CD68	514H12	Abcam, UK	1:300
CD31	1A10	Abcam, UK	1:100
ERG	EPR3864	Abcam, UK	1:1000
SOX9	EPR14335-78	Abcam, UK	1:1000
Lubricin/MSF	ab28484	Abcam, UK	1:250

**Table 4:** Aggregated SEM scores by group—separate tables cartilage and capsule.

A. Bone and Cartilage				
**Group**	**Number of Cases**	**Structural Continuity/Integrity**	**Collagen/Fibrillar Organization**	**Vascularization**
**Control**	15	0	0	0
**Pathological**	11	18	23	19
B. Capsule				
**Group**	**Number of Cases**	**Structural Continuity/Integrity**	**Collagen/Fibrillar Organization**	**Vascularization**
**Control**	15	0	0	0
**Pathological**	11	15	21	16

## Data Availability

Data are contained within the article.

## Supplementary Materials

The following supporting information can be downloaded at https://www.mdpi.com/article/10.3390/diagnostics15151932/s1: Figure S1: Semi-Quantitative IHC Scoring Examples; Table S1: Epidemiological and processing details for the pathological group (patients with advanced hip degeneration) and the control group (cadaveric specimens); Table Control Group (Cadaveric Specimens): Demographic and processing details for the control group (cadaveric specimens), including post-mortem interval (PMI), cause of death, and sample handling conditions relevant for immunohistochemical evaluation.
