# Supplementary material for: Immunohistochemical and Ultrastructural Study of the Degenerative Processes of the Hip Joint Capsule and Acetabular Labrum
Source: Diagnostics (Basel). 2025 Jul 31;15(15):1932. doi: 10.3390/diagnostics15151932 (PMC12345908; doi:10.3390/diagnostics15151932)
Supplement: Supplementary file 1 [file diagnostics-15-01932-s001.zip › Control_Group_Table Control Group.pdf]

Supplementary Table – Control Group (Cadaveric Specimens)

Table. Demographic and processing details for the control group (cadaveric specimens), including post-mortem interval (PMI), cause of death, and sample handling conditions relevant for immunohistochemical evaluation.

Specimen ID	Age (years)	Sex	Estimated PMI	Presumed Cause of Death	Fixation & Storage
C1	<40	M	6 months	Heart failure	10% formalin, stored ≥6 months before processing
C2	<40	M	6 months	Car accident	10% formalin, stored ≥6 months before processing
C3	<40	M	6 months	Car accident	10% formalin, stored ≥6 months before processing
C4	<40	M	6 months	Car accident	10% formalin, stored ≥6 months before processing
