# Supplementary material for: Immunohistochemical and Ultrastructural Study of the Degenerative Processes of the Hip Joint Capsule and Acetabular Labrum
Source: Diagnostics (Basel). 2025 Jul 31;15(15):1932. doi: 10.3390/diagnostics15151932 (PMC12345908; doi:10.3390/diagnostics15151932)

Supplementary Figure S1 – Semi-Quantitative IHC Scoring Examples

Figure S1. Representative photomicrographs illustrating the semi-quantitative immunohistochemical scoring system used in this study (scores 0–3), applied to key markers: Ki67, CD68, CD31, ERG, SOX9, and Lubricin/MSF. Each column corresponds to one specific marker, and each row to a score level. Images demonstrate typical staining patterns observed in joint capsule or labrum tissue, with increasing intensity and distribution. Magnification: 200x.

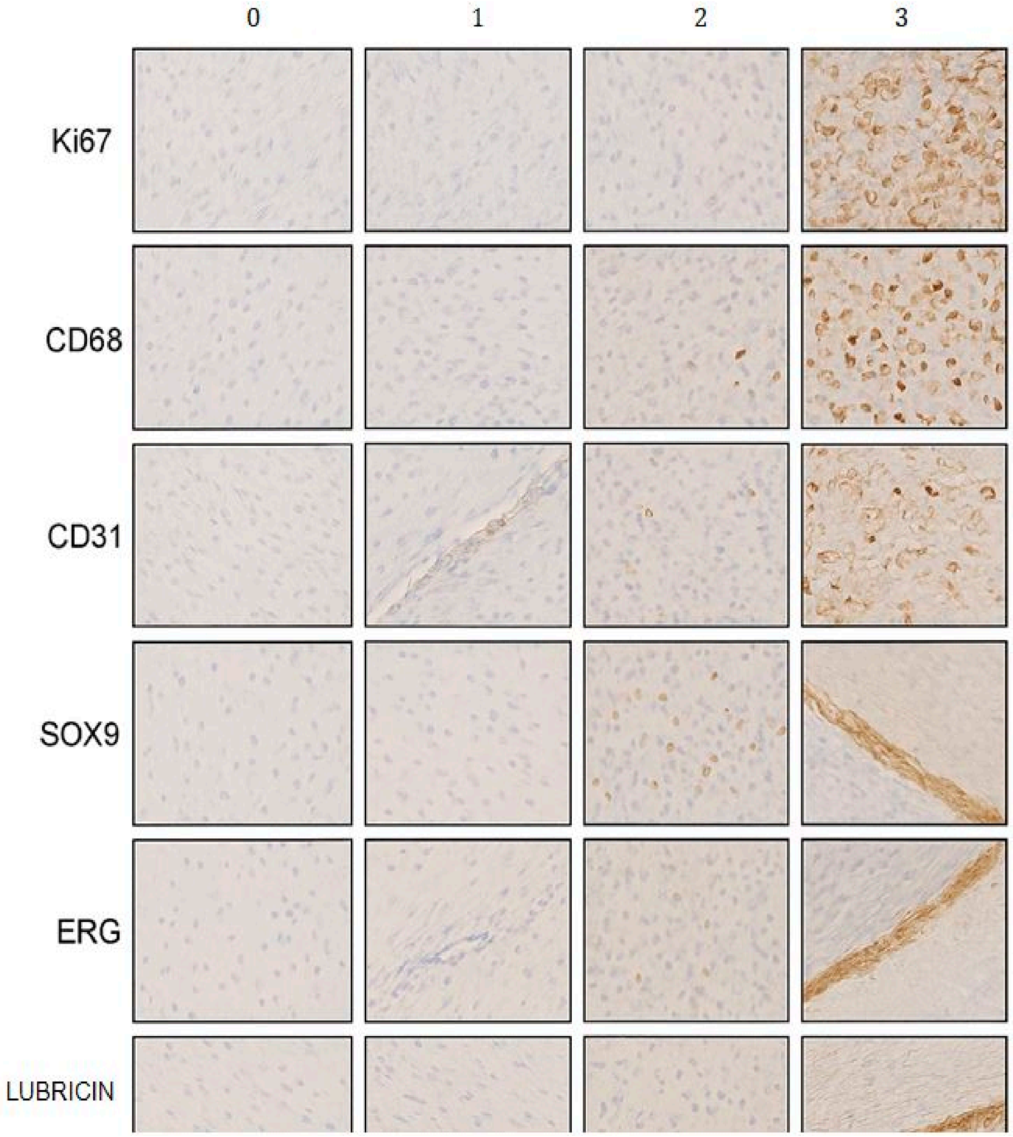

Supplement: Supplementary file 1 [file diagnostics-15-01932-s001.zip › Supplementary_Figure S1.pdf]
