# Supplementary material for: Immunohistochemical and Ultrastructural Study of the Degenerative Processes of the Hip Joint Capsule and Acetabular Labrum
Source: Diagnostics (Basel). 2025 Jul 31;15(15):1932. doi: 10.3390/diagnostics15151932 (PMC12345908; doi:10.3390/diagnostics15151932)
Supplement: Supplementary file 1 [file diagnostics-15-01932-s001.zip › Supplementary_Table S1.pdf]

Supplementary Table S1 – Demographic and Processing Data

Table S1. Epidemiological and processing details for the pathological group (patients with advanced hip degeneration) and the control group (cadaveric specimens).

Pathological Group (n = 11)

Patient ID	Age (years)	Sex	Comorbidities	Fixation & Processing
1	79	M	Hypertension, Coronary artery disease	10% formalin, processed minimum 72h
2	77	M	Chronic obliterative arteriopathy, Stroke	10% formalin, processed minimum 72h
3	75	M	Myocardial infarction, Hypertension	10% formalin, processed minimum 72h
4	68	M	Dilated cardiomyopathy, Heart failure	10% formalin, processed minimum 72h
5	70	M	Chronic coronary syndrome, Hypertension	10% formalin, processed minimum 72h
6	79	M	Hypertension, Stroke	10% formalin, processed minimum 72h
7	72	M	Arteriopathy, Coronary disease	10% formalin, processed minimum 72h
8	69	M	Heart failure, Myocardial infarction	10% formalin, processed minimum 72h
9	68	F	Hypertension, Stroke	10% formalin, processed minimum 72h
10	77	F	Dilated cardiomyopathy, Coronary artery disease	10% formalin, processed minimum 72h
11	78	F	Hypertension, Heart failure	10% formalin, processed minimum 72h
